# Role of Actin Cytoskeleton Reorganization in Polarized Secretory Traffic at the Immunological Synapse

**DOI:** 10.3389/fcell.2021.629097

**Published:** 2021-02-04

**Authors:** Victor Calvo, Manuel Izquierdo

**Affiliations:** ^1^Departamento de Bioquímica, Facultad de Medicina, Instituto de Investigaciones Biomédicas Alberto Sols, Consejo Superior de Investigaciones Científicas - Universidad Autónoma de Madrid (CSIC-UAM), Madrid, Spain; ^2^Instituto de Investigaciones Biomédicas Alberto Sols, Consejo Superior de Investigaciones Científicas - Universidad Autónoma de Madrid, Madrid, Spain

**Keywords:** T lymphocytes, B lymphocytes, immune synapse, actin cytoskeleton, protein kinase C δ, centrosome, multivesicular bodies, FMNL1

## Abstract

T cell receptor (TCR) and B cell receptor (BCR) stimulation by antigen presented on an antigen-presenting cell (APC) induces the formation of the immune synapse (IS), the convergence of secretory vesicles from T and B lymphocytes toward the centrosome, and the polarization of the centrosome to the immune synapse. Immune synapse formation is associated with an initial increase in cortical F-actin at the synapse, followed by a decrease in F-actin density at the central region of the immune synapse, which contains the secretory domain. These reversible, actin cytoskeleton reorganization processes occur during lytic granule degranulation in cytotoxic T lymphocytes (CTL) and cytokine-containing vesicle secretion in T-helper (Th) lymphocytes. Recent evidences obtained in T and B lymphocytes forming synapses show that F-actin reorganization also occurs at the centrosomal area. F-actin reduction at the centrosomal area appears to be involved in centrosome polarization. In this review we deal with the biological significance of both cortical and centrosomal area F-actin reorganization and some of the derived biological consequences.

## Introduction

**Figure 1:**
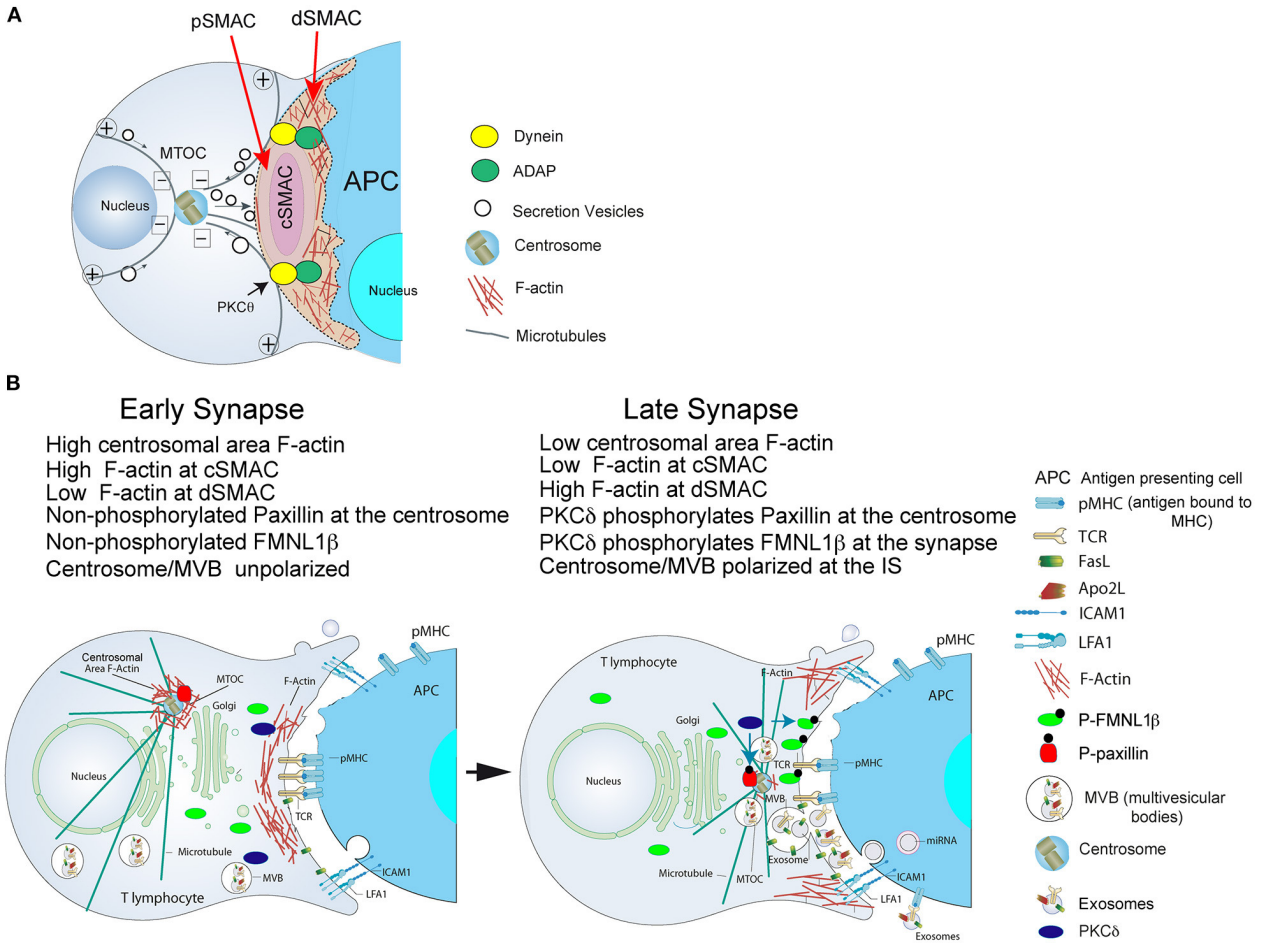
**(A)** Cortical actin cytoskeleton reorganization and MTOC polarization. After the initial scanning contact of TCR with pMHC on the APC, both naive and effector T lymphocytes form mature IS with antigen-presenting cells (APC). Th IS lasts many hours during which *de novo* cytokine (i.e., IL-2, IFN-γ) production and secretion occur, that require continuous TCR signaling. Primed effector CTL establish more transient, mature IS after scanning the target cells (i.e., a virus-infected cell), and secrete lytic granules within a few minutes. Secretory vesicles (lytic granules in CTL and cytokine-containing vesicles in Th cells) are rapidly transported (several minutes for Th cells and very few minutes or seconds for effector CTL) toward the MTOC (in the minus “–” direction) and, almost simultaneously, the MTOC polarizes toward the cSMAC of the IS, a F-actin poor area that constitutes a secretory domain. MTOC translocation to the IS appears to be dependent on PKCθ-controlled dynein anchored to ADAP at the pSMAC, that pulls MTOC in the minus direction. In both types of IS, the initial F-actin reorganization in the cell-to-cell contact area, followed by a decrease in F-actin at the cSMAC and an accumulation of F-actin at the dSMAC appears to be involved in vesicle secretion. **(B)** Actin cytoskeleton reorganization events occurring at the CD4^+^ Jurkat T lymphocyte IS model: PKCδ and MTOC/MVB polarization: both FMNL1β and paxillin are phosphorylated by PKCδ. Before forming the IS, both FMNL1β (in the cytosol) and paxillin (located at the centrosome), proteins that regulate the assembly and disassembly of F-actin, are dephosphorylated, which keeps them inactive. Left: in an early IS there is an accumulation of F-actin in the central region of the IS, while the centrosome is surrounded by a dense F-actin network that keeps it retained near the nucleus and away from the IS. Right: after PKCδ is activated by TCR stimulation at the IS, FMNL1β is phosphorylated in the C-terminal, DAD autoinhibitory domain, and is located in the IS (P-FMNL1β). In addition, paxillin is phosphorylated in Threonine 538 and remains located in the centrosome (P-paxillin). These events lead to F-actin reduction at the central region of the IS that corresponds to cSMAC, F-actin accumulation into the dSMAC and the depolymerization of F-actin surrounding the centrosome. All these processes, most probably acting in a coordinated manner, may facilitate the movement of the centrosome toward the IS and the convergence of MVB toward the F-actin depleted area in the cSMAC, which facilitates MVB fusion at the cSMAC and the subsequent secretion of exosomes (Calvo and Izquierdo, 2020) in the synaptic cleft toward the APC. For more details please refer to Herranz et al. (2019) and Bello-Gamboa et al. (2020).

T and B lymphocyte activation by antigen-presenting cells (APC) takes place at a specialized cell to cell interface called the immunological synapse (IS). IS establishment by T and B lymphocytes is a very dynamic, plastic and critical event, acting as a tunable signaling platform that integrates spatial, mechanical and biochemical signals, involved in antigen-specific, cellular and humoral immune responses (Fooksman et al., 2010; de la Roche et al., 2016). The IS is described by the formation of a concentric, bullseye spatial pattern, termed the supramolecular activation complex (SMAC), upon cortical actin reorganization (Billadeau et al., 2007; Griffiths et al., 2010; Yuseff et al., 2013; Kuokkanen et al., 2015). This reorganization yields a central cluster of antigen receptors bound to antigen called central SMAC (cSMAC) and a surrounding adhesion molecule-rich ring, called peripheral SMAC (pSMAC), which appears to be crucial for adhesion with the APC (Monks et al., 1998; Fooksman et al., 2010). Surrounding the pSMAC, at the edge of the contact area with the APC, is the distal SMAC (dSMAC), which consists of a circular array of dense filamentous actin (F-actin) (Griffiths et al., 2010; Le Floc'h and Huse, 2015) (Figure 1A).

T cell receptor (TCR) and B cell receptor (BCR) stimulation by antigen presented by APC, together with accessory molecules interaction with their ligands on the APC, induces IS formation, convergence of T and B lymphocyte secretory vesicles toward the centrosome and, almost simultaneously, centrosome polarization to the IS (Huse, 2012; de la Roche et al., 2016). This leads to polarized secretion of extracellular vesicles, lytic granules, stimulatory cytokines, or lytic proteases (Figure 1). The centrosome is the major microtubule organization center (MTOC) in T and B lymphocytes, and consists of two centrioles surrounded by pericentriolar material (PCM), generating a radial organization of microtubules (Sanchez and Feldman, 2017). IS formation is associated with an initial increase in cortical F-actin at the IS (Billadeau et al., 2007), followed by a decrease in F-actin density at the central region of the IS that includes the cSMAC and contains the secretory domain (Griffiths et al., 2010; Ritter et al., 2015). Subsequently, F-actin recovery at the cSMAC leads to conclusion of lytic granule secretion in cytotoxic T lymphocytes (CTL) (Ritter et al., 2017). These reversible actin cytoskeleton reorganization processes occur during lytic granule secretion in CTL and cytokine-containing vesicle secretion in T-helper (Th) lymphocytes (Na et al., 2015; Ritter et al., 2017).

## Immune Synapse Morphology Formed by Different Immune Cells

The IS has long been characterized by the general concentric architecture that adopts during its maturation (Le Floc'h and Huse, 2015). The bullseye actin cytoskeleton architecture of the IS and the F-actin reorganization process are common to CD4^+^ Th lymphocytes, CD8^+^ CTL, B lymphocytes, and natural killer (NK) cells forming IS (Rak et al., 2011; Lagrue et al., 2013; Le Floc'h and Huse, 2015). However, for space reasons in this review we deal only with IS made by T and B lymphocytes. The bullseye pattern of a mature synapse includes redistribution of F-actin and surface receptors in concentric regions. In this context, radially symmetric spreading of the T lymphocyte over the surface of the APC is conducted by protrusive actin polymerization (Le Floc'h and Huse, 2015; de la Roche et al., 2016), leading to TCR-pMHC interactions at the tip of these actin-rich interdigitations (de la Roche et al., 2016). This is accompanied by the formation of TCR microclusters in the synaptic membrane that, as TCR signaling is initiated, coalesce in the center of the synapse to form the cSMAC (Le Floc'h and Huse, 2015; de la Roche et al., 2016). Thus, as the IS develops, retrograde (centripetal) F-actin flow drives TCR microclusters into the cSMAC, while F-actin intensively reorganizes into the peripheral ring that will become the dSMAC (Le Floc'h and Huse, 2015). F-actin flow within the dSMAC promotes adhesion by clustering integrins such as LFA-1 in the pSMAC (Comrie et al., 2015), that eventually will interact with ICAM-1 located on the APC (Figure 1B), reinforcing adhesion between these cells and amplifying TCR signaling (Le Floc'h and Huse, 2015; de la Roche et al., 2016). Remarkably, all the above mentioned immune cells share the capability to directionally secrete stimulatory cytokines, proteases, cytotoxic factors, or extracellular vesicles (including exosomes) at the IS (Calvo and Izquierdo, 2020). This F-actin structure for polarized secretion is thought to enhance the specificity and efficiency of the triggered biological responses (Le Floc'h and Huse, 2015). Although the IS formed by all these immune cells share this general F-actin pattern, important differences in terms of stability, duration but also synaptic F-actin structure exist among CD4^+^ Th, CD8^+^ CTL, B lymphocytes, and NK cells (Murugesan et al., 2016; Carisey et al., 2018). For instance, whereas CD4^+^ cells form stable IS (from minutes up to several hours), that are necessary for both directional and continuous secretion of stimulatory cytokines (Ueda et al., 2011), IS made by primed CTL trigger the rapid polarization (from seconds to very few minutes) of their lytic granules or secretory lysosomes (SL) toward the IS (Griffiths et al., 2010; Huse, 2012). CTL form very transient IS, lasting only few minutes, until target cells are eliminated. Optimal CTL function is thought to require rapid and transient contacts in order to consecutively deliver as many successive lethal hits as possible to several target cells (Calvo and Izquierdo, 2020). Apart from these kinetic and stability differences, the canonical bullseye actin pattern was described in CD4^+^ T lymphocytes using lipid bilayers or upon interaction with B cells. However, a variation of this canonical pattern was observed when CD4^+^ T lymphocytes were challenged with dendritic cells as APC, resulting in a multifocal cell to cell IS (Brossard et al., 2005; Kumari et al., 2019). Thus, 3D spatial differences between the IS made by the same immune cell type exist. Moreover, under comparable stimulation conditions, differences in actin cytoskeleton spatial organization and dynamics among immortalized human and primary mouse and human T lymphocytes exist (Colin-York et al., 2019; Kumari et al., 2019), and there are differences in the organization and molecular mechanisms underlying these F-actin networks (Kumari et al., 2019) (Table 1).

**Table 1:** F-actin reorganization and polarized secretion events in T and B lymphocyte IS and involved proteins.

	**T lymphocyte IS**		**B lymphocyte IS**	
(*)	**CTL/APC**	**CD4^**+**^ T cell /APC**	**B/APC**	**B /CD4^**+**^ T cell**
F-actin reorganization at the IS	+ CDC42/WASP/ARP2/3(Billadeau et al., 2007; Sinai et al., 2010) TAGNL2 (Na et al., 2015)	+ CDC42/WASP/ARP2/3 (Chemin et al., 2012) dynamin 2 (Gomez et al., 2005) CDC42 (Stowers et al., 1995) FMNL1, Dia1 (Murugesan et al., 2016) HS1 (Gomez et al., 2006) TAGNL2 (Na et al., 2015)	+ CDC42/WASP/ARP2/3 Ezrin, Moesin(Kuokkanen et al., 2015)	+ TAGNL2(Na et al., 2016)
F-actin reduction at cSMAC and centrosome polarization	+ (Ritter et al., 2015, 2017) CDC42/IQGAP1 (Stowers et al., 1995; Stinchcombe et al., 2006)	+ PKCδ, FMNL1β(Herranz et al., 2019; Bello-Gamboa et al., 2020) Dynein(Combs et al., 2006; Liu et al., 2013; Sanchez et al., 2019)	+ dynein, proteasome(Schnyder et al., 2011; Ibanez-Vega et al., 2019)	Unknown
F-actin reduction at centrosome and centrosome polarization	Unknown	+ PKCδ, paxillin (Bello-Gamboa et al., 2020) WASH, ARP2/3(Farina et al., 2016)	+ ARP2/3(Obino et al., 2016) Proteasome(Ibanez-Vega et al., 2019)	Unknown
Lytic granules and/or Exosome secretion	+ (Peters et al., 1989)	+ (Alonso et al., 2011)	+ (Yuseff et al., 2011; Kuokkanen et al., 2015)	+ (Muntasell et al., 2007)
Centrosome polarization	+ (Stinchcombe et al., 2006)	+ (Ueda et al., 2011)	+ (Yuseff et al., 2011)	+ (Duchez et al., 2011)
Mechanisms of centrosome polarization	Paxillin (Herreros et al., 2000; Robertson and Ostergaard, 2011) FMNL1, Dia1 (Gomez et al., 2007) CDC42/IQGAP1 (Stowers et al., 1995; Stinchcombe et al., 2006)	FMNL1, Dia1 (Gomez et al., 2007) PKCθ, dynein (Quann et al., 2009) CDC42 (Stowers et al., 1995) PKCδ, paxillin(Herranz et al., 2019; Bello-Gamboa et al., 2020)	ARP2/3 (Obino et al., 2016) Proteasome(Ibanez-Vega et al., 2019)	Unknown
DAG/DGK-control of centrosome polarization.	+ (Quann et al., 2009)	+ DAG, DGKα(Quann et al., 2009; Alonso et al., 2011) DAG, dynein(Liu et al., 2013) DAG/dynein/PKC (Sanchez et al., 2019)	+ DAG, DGKζ (Merino-Cortés et al., 2020)	Unknown
PKC/PKD control of secretory granules/MVB traffic	+ PKCδ (Ma et al., 2007) PKCθ(Monks et al., 1998; Quann et al., 2011)	+ PKCδ (Herranz et al., 2019) PKD1/2 (Mazzeo et al., 2016)	+ PKCζ (Siemasko et al., 1998; Yuseff et al., 2011) PKD1/3 (Mazzeo et al., 2016)	Unknown

(*) The quoted biological response in the first column corresponds to the response of the effector, first cell type for each indicated cell-cell synapse.

Although IS architecture and dynamics are major determinants of antigen recognition and signaling by TCR and BCR, the molecular components that contribute to the distinct F-actin patterns observed in IS formed by different immune cells remain largely unknown (Kumari et al., 2019). Indeed, this knowledge is required to understand how different immune cells acquire and develop their functional specialization. It has been speculated that the actin cytoskeleton can arbitrate a force balance across the IS interface to regulate the dimensions and lifetimes of diverse subsynaptic zones that, in turn, may alter different T cell activation steps (Kumari et al., 2019). Remarkably, several F-actin regulatory proteins are different for each synapse subtype (summarized in Table 1), thus these differences may underlie the spatiotemporal differences in F-actin architecture existing among different IS. It is out of the scope of this review to detail these differences, please refer to some excellent reviews on this subject, that include also data on F-actin reorganization in the IS made by NK cells (Billadeau et al., 2007; Lagrue et al., 2013; Hammer et al., 2018; Li et al., 2018; Blumenthal and Burkhardt, 2020).

## Signals Regulating Cortical Actin Reorganization in the Immune Synapse

cSMAC, pSMAC, and dSMAC formation characterizes a mature IS and is the basis of a signaling platform that integrates signals and coordinates molecular interactions leading to both exocytic and endocytic processes, necessary for an appropriate antigen-specific immune response (Griffiths et al., 2010; Xie et al., 2013). Actin reorganization plays a central role in IS maintenance, as well as in antigen receptor-derived signaling (Billadeau et al., 2007). In brief, TCR and BCR stimulation, together with interaction of adhesion and co-stimulatory molecules at the IS, trigger early second messengers such as calcium and diacyglycerol (DAG) (Izquierdo and Cantrell, 1992). DAG regulates several protein kinase C (PKC) family members (including novel PKC members, PKCδ and PKCθ), protein kinase D (PKD1), and Ras/ERK2 pathway (Spitaler et al., 2006), leading to the activation of the two major actin regulatory pathways: the formin (FMNL1 and Dia) pathway involved in F-actin nucleation, and the CDC42/WASP/ARP2/3 pathway involved in actin filament branching (Kühn and Geyer, 2014; Kumari et al., 2014). In the IS made by T lymphocytes, TCR engagement triggers multiple signaling pathways that regulate actin organization and rearrangements at the different F-actin networks inside and outside the IS (Billadeau et al., 2007; Kumari et al., 2014; Le Floc'h and Huse, 2015; Hammer et al., 2018). Thus, actin polymerization is a result of complex molecular interactions, and a diverse and complex set of TCR-downstream molecular pathways contributes to actin polymerization. Once the T cell is bound to the pMHC complex on the APC, the immunoreceptor tyrosine-based motifs (ITAM) in the cytosolic tail of the TCR-associated CD3 complex undergo phosphorylation, which then serve as docking sites for the Syk-kinase zeta chain-associated protein of 70 kDa (ZAP70) (Kumari et al., 2014). CD3-recruited ZAP70 then leads to phosphorylation and activation of two key adaptor molecules that associate with a variety of molecules in TCR-associated signaling complexes. First, ZAP70 phosphorylates linker activation of T cells (LAT), which in turn associates via Gads with the SH2 domain-containing leukocyte protein of 76 kDa (SLP76). SLP76 recruitment to phosphorylated LAT allows subsequent SLP76 phosphorylation by ZAP70. Phosphorylated LAT also directly interacts with PLCγ1, a major regulator of Ca2+ influx in response to TCR triggering. PLCγ is recruited via LAT interaction to the synaptic membrane and generates DAG at the IS. SLP76 acts as a scaffold for a multitude of actin effectors including Rho GTPases nucleotide exchange factor, Vav1, adaptor molecule Nck, and actin nucleation promoting factors (NPFs). Alternatively, Rho GTPases (CDC42, Rho, Rac) can also bind and activate actin NPFs (ARP2/3, WAVE), which in turn activate actin nucleation factors (ARP2/3, formins), ultimately leading to F-actin formation. In addition, PI3K activated by LAT/SLP76 activation produces PIP_3_ at the plasma membrane that, by recruiting DOCK2 to the IS, activates Rac and, ultimately, WAVE/ARP2/3 (Le Floc'h and Huse, 2015). An intriguing feature of actin cytoskeleton regulation at the IS is the variety of both actin NPFs and actin nucleation factors involved in actin cytoskeleton assembly. This could be related to the complexity of the four F-actin networks contributing to the architecture of the IS described below (Hammer et al., 2018).

More recently, by using super-resolution imaging techniques, such as total internal fluorescence microscopy combined with 3D structured-illumination microscopy (TIRFM/3D-SIM) on functionalized stimulatory surfaces, it has been shown that following initial TCR-antigen interaction, at least four discrete F-actin networks form and maintain the shape and function of this canonical IS (Hammer et al., 2018; Blumenthal and Burkhardt, 2020): branched F-actin network at the dSMAC controlled by WAVE/ARP2/3 activity; actomyosin arc network at the pSMAC controlled by formin Dia1 and phosphorylated myosin; hypodense F-actin at the cSMAC controlled by ARP2/3 and probably formins such as FMNL1/Dia1; F-actin foci at the dSMAC and pSMAC controlled by WASP and HS1 (Hammer et al., 2018; Blumenthal and Burkhardt, 2020). Therefore, in the same cell type distinct regulators control discrete F-actin networks. The four F-actin networks at the T lymphocyte IS are related to the three distinct functional and signaling areas at the IS (SMACs). Thus, the outer ring of the IS, the dSMAC, corresponds to the lamellipodium region of a migrating cell and forms quickly upon TCR stimulation and contains highly-branched actin filaments generated by the ARP2/3 activator WAVE2. Radially arranged within the dSMAC are bundles of linear actin filaments, generated by formin (Dia) activity near the edge of the spreading lymphocyte, that bend as they move inward the dSMAC, forming actomyosin arcs spanning the pSMAC. Therefore, these actomyosin arcs define the pSMAC, which is enriched in integrins. Actomyosin network disassembly at the pSMAC leads to a F-actin poor region in the center of the IS known as the cSMAC. cSMAC is associated with receptor internalization and provides a site for exocytic vesicle secretion (Figure 1). Finally, the fourth actin network consists of dense actin foci related to protrusive structures rich in F-actin called invadosome-like protrusions (ILPs) (Hammer et al., 2018; Blumenthal and Burkhardt, 2020). Recent evidences support the view that the described F-actin network complexity is, at least in part, based on the action of different F-actin regulatory pathways. For instance, branched F-actin network at the dSMAC is controlledby PI3K-PIP3-DOCK2-WAVE/ARP2/3, whereas F-actin foci at the cSMAC is regulated by ZAP70-LAT/SLP76-VAV-CDC42-WASP-ARP2/3 (Hammer et al., 2018).

## Synaptic Actin Cytoskeleton Control of Centrosome and Secretion Vesicles Polarization: Release of Extracellular Vesicles and Lytic Granules

Regarding the mechanisms controlling centrosome polarization and the role of cortical actin reorganization in MTOC polarization, DAG production at the IS has been shown to be important for centrosome polarization in both CTL and CD4^+^ T lymphocytes forming IS (Quann et al., 2009). With respect to potential DAG effectors, DAG-activated PKCθ at the IS triggers the adhesion and degranulation-adaptor protein (ADAP)/dynein complex localization at the F-actin/integrin rich, pSMAC ring (Figure 1A). Since dynein is a minus end-directed microtubule motor, after being recruited to the IS it can bind microtubules and reorient the centrosome by minus end-directed motion (Combs et al., 2006; Quann et al., 2009, 2011; Liu et al., 2013) (Figure 1A). At the early stages of IS formation F-actin accumulates at the lymphocyte-APC contact area to generate filopodia and lamellipodia, that produce dynamic changes between extension and contraction in the lymphocyte over the APC surface (Le Floc'h and Huse, 2015). Subsequently, once IS growth has stabilized, cortical F-actin accumulates into the dSMAC, and F-actin reduction at the cSMAC appears to facilitate secretion toward the APC by focusing secretion vesicles on the IS (Stinchcombe et al., 2006), creating regions within the canonical bullseye IS structure (Figure 1A). Thus, F-actin reduction at the cSMAC does not simply allow secretion, since it apparently plays an active role in centrosome movement to the IS (Stinchcombe et al., 2006; Ritter et al., 2015; Sanchez et al., 2019). It is widely thought that centrosome reorientation promotes cytotoxic and Th lymphocyte specificity by guiding secretory vesicles to the IS for directional secretion (Stinchcombe et al., 2006; Huse et al., 2008). However, in the context of secretory traffic at the IS, not always centrosome polarization is necessary for either secretory vesicle transport or secretion at the IS in CD4^+^ T lymphocytes (Chemin et al., 2012) or lytic granule polarization and secretion in CTLs (Ma et al., 2007; Bertrand et al., 2013; Nath et al., 2016). For instance, it has been shown that an early and rapid secretion phase of lytic granules constitutively positioned nearby the IS precedes centrosome polarization at the CTL-target cell IS (Bertrand et al., 2013). In addition, in PKCδ-KO mouse CTL, lytic granules did not polarize to the IS and, subsequently, CTL activity and target cell death were inhibited (Ma et al., 2007). However, centrosome polarization toward the IS was not affected by the absence of PKCδ (Ma et al., 2007). In contrast, in the synapses made by CD4^+^ Jurkat T lymphocytes, multivesicular bodies (MVB, a type of secretory vesicles involved in exosome secretion by CD4^+^ Jurkat and primary CD8^+^ and CD4^+^ T lymphocytes (Alonso et al., 2005, 2011)) and centrosome always co-migrated toward the IS, and MVB and centrosome did not polarize in PKCδ-interfered CD4^+^ Jurkat T lymphocytes and the centrosome/MTOC center of mass (MTOC^C^) was coincident or very proximal to the MVB center of mass (MVB^C^) regardless of polarization (Herranz et al., 2019; Bello-Gamboa et al., 2020). In summary, all these results broaden current views of CTL biology by revealing an extremely rapid lytic granule secretion step and by showing that centrosome polarization is dispensable for efficient lytic granule secretion. All these examples of segregation between centrosome movement and lytic granule polarization point out that centrosome repositioning, secretory vesicles traffic, and F-actin architecture and dynamics at several locations should be analyzed at the single cell level, to obtain a more complete view of the secretion process.

In addition, it has been recently described that centriole-deficient CTL exhibited reduced cytotoxicity due to an alteration in secretory granule biogenesis, although this deficient response was not due to impaired polarized secretion, since lytic granule traffic and secretion toward the IS remained unaffected (Tamzalit et al., 2020). Instead, it has been proposed that the defect was in part due to impaired F-actin reorganization at the IS produced by centriole deletion (Tamzalit et al., 2020), which points out an unexpected role of the intact centrosome in supporting synaptic F-actin architecture and dynamics. Interestingly, CTL lacking centriole formed synapses that lacked an obvious F-actin cleared region at the cSMAC, a similar phenotype to that found in PKCδ-interfered CD4^+^ Jurkat T lymphocytes (Herranz et al., 2019; Bello-Gamboa et al., 2020). Polarized secretion is known to occur in IS domains that have been cleared of F-actin and are, thus, accessible to secretion vesicles (Griffiths et al., 2010; Huse, 2012; Ritter et al., 2015; Herranz et al., 2019).

More recently, it has been shown that PKCδ-dependent F-actin clearing at the cSMAC and PKCδ-dependent phosphorylation of formin FMNL1β at the IS, are involved in centrosome/MVB polarization leading to exosome secretion in CD4^+^ Jurkat T lymphocytes forming IS (Herranz et al., 2019; Bello-Gamboa et al., 2020). The formins Dia and FMNL1 are constitutively inactive because they undergo intramolecular, autoinhibitory folding in the cytoplasm, which blocks their ability to nucleate and elongate actin filaments (Hammer et al., 2018). In this context, a different formin, FMNL2, is phosphorylated by PKCα and PKCδ at S1072, reversing its autoinhibition by the C-terminal, DAD auto-inhibitory domain and enhancing F-actin assembly, β1-integrin endocytosis, and invasive motility (Wang et al., 2015). In the FMNL1 isoform FMNL1β, S1086 is surrounded by a sequence displaying high homology to the one surrounding S1072 of FMNL2 (Wang et al., 2015; Bello-Gamboa et al., 2020). Our results support that IS-induced, PKCδ-dependent phosphorylation in FMNL1β C-terminal region containing the auto-inhibitory domain (possibly at S1086) activates FMNL1β and mediates centrosome polarization. Thus, PKCδ appears to regulate F-actin reorganization, most probably, by controlling FMNL1β activation through phosphorylation at S1086 (Bello-Gamboa et al., 2020), as certain PKC isoforms activate FMNL2 activity (Wang et al., 2015).

## Centrosomal F-actin and Centrosome Polarization

The centrosome nucleates and anchors microtubules and is thus considered to be the principal MTOC. In addition, few years ago it was discovered that the centrosome organizes a local F-actin network and should be considered as a F-actin organizing center (Farina et al., 2016). Isolated centrosomes from Jurkat T lymphocytes efficiently nucleate actin filaments, and the centrosome is associated *in vivo* with an actin network, and these results were extended to other cell types (Farina et al., 2016; Plessner et al., 2019). Moreover, actin filament nucleation at the centrosome is mediated by the nucleation-promoting factor Wiskott-Aldrich syndrome protein (WASP) and SCAR homolog (WASH), in combination with the ARP2/3 complex (Farina et al., 2016). Pericentriolar material seems to modulate F-actin network by regulating WASH/ARP2/3 recruitment to the centrosome (Farina et al., 2016).

### Centrosomal F-actin and B Lymphocytes

Centrosome-associated ARP2/3 locally nucleates F-actin, which is needed for centrosome tethering to the nucleus (Obino et al., 2016). Upon B lymphocyte activation with BCR-ligand-coated beads as a synapse model, ARP2/3 is partially depleted from the centrosome, as a result of its recruitment to the IS, where it regulates cortical F-actin. This leads to a reduction in F-actin nucleation at the centrosome and thereby allows its detachment from the nucleus and polarization to the IS (Obino et al., 2016). Thus, centrosomal F-actin depletion appears to be crucial allowing centrosome polarization toward the IS during BCR stimulation in B lymphocytes (Obino et al., 2016). Thus, both *in vitro* and living-cell experiments support this new view of centrosome as a genuine and plastic F-actin-organizing center. However, the precise function of the F-actin network at the centrosome is not well understood. In the same B lymphocyte model, F-actin depletion around the centrosome, F-actin reorganization at the IS, and centrosome polarization depend on proteasome activity (Ibanez-Vega et al., 2019). By inhibiting proteasome activity, an inhibition of F-actin dismantling around centrosome correlated with the inhibition of centrosome polarization toward the B lymphocyte synapse (Ibanez-Vega et al., 2019). Thus, it appears that at least two mechanisms controlling centrosomal area F-actin co-exist in B lymphocytes, and both regulate centrosome polarization.

### Centrosomal F-actin and T Lymphocytes

We have shown that F-actin clearing at the cSMAC and centrosomal area F-actin depletion, respectively, mediated by PKCδ-dependent phosphorylation of FMNL1β or paxillin, are associated with centrosome/MVB polarization and exosome secretion in CD4^+^ Jurkat T lymphocytes forming IS (Herranz et al., 2019; Bello-Gamboa et al., 2020) (Figure 1B). Although PKCδ appeared to regulate centrosomal area F-actin, FMNL1β did not appear to participate in this regulation (Bello-Gamboa et al., 2020). A possible PKCδ downstream effector involved in centrosomal area F-actin reorganization could be the actin regulatory protein paxillin, whose phosphorylation at threonine 538 (T538) by PKCδ leads to actin cytoskeleton depolymerization and regulates integrin-mediated adhesion and migration of B lymphoid cells (Romanova et al., 2010). Moreover, the centrosome cannot polarize to the IS in paxillin-interfered CTL (Robertson and Ostergaard, 2011). In addition, paxillin phosphorylation is required for CTL lytic granule secretion (Robertson et al., 2005), and both paxillin (Herreros et al., 2000) and PKCδ (Fanning et al., 2005) are localized at the centrosome. Thus, in CD4^+^ Jurkat T lymphocytes forming IS, we have found that PKCδ-dependent paxillin phosphorylation may govern a F-actin reorganization network different from F-actin at the IS, such as centrosomal F-actin, that may also contribute to the diminished centrosome polarization observed in PKCδ-interfered Jurkat T lymphocyte clones (Figure 1B). This PKCδ-dependent, paxillin-regulated mechanism for centrosomal area F-actin reorganization appears to co-exist in CD4^+^ Jurkat T lymphocytes with the PKCδ-dependent, FMNL1β-regulated mechanism for cortical F-actin reorganization explained above. More research is necessary (i.e., experiments involving phospho-deficient and phospho-mimetic mutants of paxillin at T538 and/or FMNL1β at S1086) to establish the relative contribution of these mechanisms to the polarization processes.

In addition, impaired F-actin reorganization at the IS was produced by centriole deletion in CTL (Tamzalit et al., 2020), which points out an unexpected role for the intact centrosome and/or centrosomal F-actin in supporting synaptic F-actin architecture and dynamics. Moreover, lower centrosomal actin filament densities enhanced microtubule growth at the centrosome (Inoue et al., 2019), that decisively affected cell adhesion and spreading. These results, together with the fact that ARP2/3 is partially depleted from the centrosome as a result of its recruitment to the IS (Farina et al., 2016), suggest an unsuspected direct or indirect interaction (i.e., competition for F-actin regulators such as ARP2/3), between cortical and centrosomal area F-actin networks, that in turn may regulate tubulin cytoskeleton at different subcellular locations. The distinct F-actin networks may be functionally interconnected by coordinated activation of different actin assembly factors (Bello-Gamboa et al., 2020) (Figure 1B), competition for the same regulatory factor (Obino et al., 2016) and/or actin monomer availability (Suarez and Kovar, 2016). Pericentriolar material seemed to modulate the centrosomal F-actin network by regulating ARP2/3 and WASH recruitment to the centrosome (Farina et al., 2016). Since F-actin reorganization at the remaining proteinaceous pericentriolar material (PCM) area, containing pericentrin and γ-tubulin, was not analyzed in centriole-deficient CTL (Tamzalit et al., 2020), it will be interesting to study pericentriolar area F-actin in detail in these cells. In addition, considering that PKCδ is located in the centrosome (Fanning et al., 2005; Ma et al., 2008a), but also in lytic granules in CTL (Ma et al., 2008b), it is conceivable that PKCδ, directly or indirectly, may coordinately regulate both centrosomal area and synaptic F-actin networks.

## Current Research Gaps

### Lipid Bilayer Synapse Model

Most of what we know about the formation, organization, and dynamics of the four described F-actin and actomyosin networks at the IS results from high spatio-temporal resolution image analysis of T cells engaged with activating surfaces such as coated glass and planar lipid bilayers, which position these networks in the ideal imaging plane, avoiding the Z spatial dimension (Hammer et al., 2018). This approach is certainly somewhat reductionist since it is not possible to guarantee that all the molecular interactions occurring in a real, cell to cell synapse will also occur upon interaction with the coated glass or the lipid bilayer (Fooksman et al., 2010). In fact, supported lipid bilayers do not completely imitate the complex and irregular surface of an APC or target cell, possibly causing non-physiological interactions in the IS (Bertolet and Liu, 2016). Thus, although studies using supported planar bilayers are powerful in terms of resolution and sensitivity, it is important to test the predictions of these model systems using *in vitro* or *in vivo* cell–cell systems in order to extend the results to a more physiologic scenario (Dustin, 2009).

### Actin Cytoskeleton in Primary vs. Immortalized T Cells and Different Synapse Subtypes

Striking differences in F-actin architecture and dynamics at the IS have been found between human primary CD4^+^ and immortalized CD4^+^ T lymphocytes, such as Jurkat cells, under comparable activation conditions on lipid bilayers (Colin-York et al., 2019; Kumari et al., 2019). In contrast, no major differences were found between the synaptic architecture of Jurkat cells and mouse CD8^+^ primary CTLs (Murugesan et al., 2016) on stimulatory lipid bilayers. F-actin network dynamics and mechanics are likely to be different in these cells, and influence how T cells employ mechanical cues in different ways during antigen recognition (Kumari et al., 2019). Thus, caution should be taken when generalizing the cellular mechanisms underlying the variety of IS patterns and motility behaviors to specific T cell subtypes and across different species (Kumari et al., 2019). It is conceivable that these differences also exist in different real cell-cell synapses, although they have not been observed yet (Colin-York et al., 2019; Kumari et al., 2019). In addition, while PKCδ regulates centrosomal area F-actin upon IS formation by CD4^+^ Jurkat cells (Bello-Gamboa et al., 2020), more research is necessary to extend these results to both CD8^+^ and CD4^+^ primary T lymphocytes forming IS. While TIRFM combined with super-resolution 3D-SIM provided probably the best live-cell images of synapses on lipid bilayer models (Murugesan et al., 2016; Colin-York et al., 2019), the best live-cell images of F-actin architecture and dynamics within T cells engaged with an APC were obtained using high temporal resolution, lattice light sheet microscopy (LLSM) in a diffraction-limited mode (Ritter et al., 2015; Fritzsche et al., 2017). An option to improve the image spatial resolution is taking advantage of the better XY resolution of microscopes with respect to the axial (Z) resolution (Calvo and Izquierdo, 2018), by using LLSM combined with 3D-SIM. Other options are placing the T lymphocyte on top of an APC by using “pairing and coupling” microfluidic devices (Jang et al., 2015), or on a very flat APC in which the IS is located in the XY optical plane (Wang et al., 2018). These last two options will indeed provide real cell-cell synapse models. While LLSM images in a diffraction-limited mode (Ritter et al., 2015; Fritzsche et al., 2017) showed with remarkable definition the branched F-actin network dynamics in the dSMAC, including its contribution to a flow of F-actin up the sides of the APC-bound T cell, none of the four F-actin networks observed by TIRFM combined with 3D-SIM in lipid bilayers were visible using LLSM (Hammer et al., 2018). These latter F-actin networks, all of which are much fainter F-actin structures than those observed in the dSMAC (Hammer et al., 2018), will probably become discernible in the future by using one or more of the cell setups and super-resolution imaging methods described above.

### Centrosomal F-actin Network Characterization and Measurements

Although in the original publication the authors defined the existence of a “centrosomal” F-actin network, it should be underlined this is an operative definition that does not specify the extension and/or the limits of such a network (Farina et al., 2016). In fact, the authors arbitrarily defined a 2 μm-diameter region of interest (ROI) including the centrosome for F-actin fluorescence intensity measurements. Subsequent publications have incorporated for centrosomal F-actin measurements the use of a 1.6–2 μm diameter, centrosome-centered ROI (Obino et al., 2016; Ibanez-Vega et al., 2019; Bello-Gamboa et al., 2020). However, a more appropriate term could be “centrosomal area” F-actin, to remark that measures were referred to an area centered at the centrosome and surely larger than the centrosome itself. Indeed, this is a more accurate term that includes the complexity of organelle interconnection. This is an important issue for centrosomal area F-actin evaluation, since the imaging techniques used in these publications (confocal microscopy, TIRFM, epifluorescence microscopy plus deconvolution) do not allow enough resolution to sustain that F-actin assembles at the pericentrosomal matrix instead of other membrane-bound organelles included in this area (Obino et al., 2016; Bello-Gamboa et al., 2019; Ibanez-Vega et al., 2019). Since interphase centrosomes are smaller than mitotic centrosomes (Decker et al., 2011) and cells may be at any cell cycle phase, it is difficult to establish a fixed centrosomal diameter. If a 2 μm-diameter centrosomal area ROI is analyzed, it cannot be ruled out that other F-actin-regulating organelles are included in this area, such as the Golgi, endosomes, or MVB (Colon-Franco et al., 2011; Bello-Gamboa et al., 2020). MVB are also involved in actin polymerization at the IS during intracellular reorganization (Calabia-Linares et al., 2011). Electron microscopy images show that vesicles and endosomes are located nearby the centrosomes (Ueda et al., 2011). The use of a pericentriolar marker, together with new imaging super-resolution techniques, would facilitate the study of the centrosomal area and the specific localization and dynamics of centrosomal F-actin. Indeed, in the future, emerging and promising techniques, such as LLSM (Ritter et al., 2015; Fritzsche et al., 2017), combined with non-diffraction limited, super-resolution microscopy (Fritzsche et al., 2017; Calvo and Izquierdo, 2018), may help to a better definition of centrosomal area F-actin structure and function, as it occurred for the four recently defined, synaptic F-actin networks that contribute to maintain the shape and function of the canonical IS (Section Signals Regulating Cortical Actin Reorganization In The Immune Synapse). In this context, TIRFM or TIRFM combined with 3D-SIM are exceptionally useful imaging techniques to study both F-actin cytoskeleton and secretion vesicle degranulation on the XY plane of coated glass or lipid bilayer (Rak et al., 2011; Murugesan et al., 2016; Sinha et al., 2016; Carisey et al., 2018), due to its high signal-to-noise ratio and improved spatial resolution below the diffraction limit (Calvo and Izquierdo, 2018). However, this procedure is somewhat limited since only an illuminated homogeneous surface can be used to stimulate cells and TIRFM would dismiss subcellular structures (i.e., centrosome) or molecules located beyond a minor Z distance from the stimulatory surface (>200–300 nm). Thus, centrosome movement in the Z dimension from distal subcellular locations cannot be imaged, although its last stages approaching the IS can be properly imaged. One possibility to circumvent this problem is to limit centrosome movement in the Z dimension by using the cell setups explained above. Indeed, other techniques as the already mentioned diffraction-limited LLSM, combined with 2D or 3D stimulated emission depletion (STED) super-resolution microscopy (Fritzsche et al., 2017), may provide in the future new tools to address the study of centrosomal area F-actin organization and dynamics at the nanoscale level.

## Potential Future Developments in the Field. Imaging the Immunological Synapse

For adequate IS imaging by fluorescence microscopy, harmonizing temporal and spatial resolutions, overcoming spatial constraints due to imaging in Z optical axis, improving signal-to-noise ratio, and solving the photobleaching and cytotoxicity inherent to any live cell imaging, are required (Combs and Shroff, 2017; Calvo and Izquierdo, 2018). Please refer to some excellent reviews dealing with the more relevant fluorescence microscopy techniques used in cell biology in general (Combs and Shroff, 2017; Lambert and Waters, 2017; Sahl et al., 2017), and specifically for IS imaging (Rossy et al., 2013; Calvo and Izquierdo, 2018; Herranz et al., 2019), since it is out of the scope of this review to deal with these relevant technical issues. In this context, it is remarkable that to date difficulties in visualization of both primary and transformed T lymphocyte models at high spatio-temporal resolution have somewhat limited our understanding of the principles underlying T lymphocyte subtype-specific activation (Kumari et al., 2019). However, visualization and quantification of actin cytoskeleton and the underlying patterns and dynamics has evolved to a significant degree due to the advances in microscopy regarding spatiotemporal resolution (Kumari et al., 2019). Currently, we only have a limited knowledge of how actin cytoskeleton organization affects distinct synaptic patterning and signaling. This is due to several facts, including the low transfection efficiency of F-actin reporters in primary lymphocytes, the rapid kinetics of changes, the reduced area of cell-cell synapses, and the active, highly plastic and irregular cell-cell synaptic interface that precludes image capture at high spatiotemporal resolution (Kumari et al., 2019; Blumenthal and Burkhardt, 2020). In the future, the development of emerging and promising techniques such as LLSM (Ritter et al., 2015) and new 3D live-cell super-resolution microscopy (Fritzsche et al., 2017), combined with some useful probes for F-actin in living cells (Lukinavicius et al., 2013, 2014; Bello-Gamboa et al., 2020) will transform how we image cellular and protein dynamics during IS interactions. These advances will indeed shed more light into our knowledge of these processes.

Thus, some techniques of choice have been specifically used for IS imaging and to overcome the mentioned caveats. Planar lipid bilayers and coverslips or beads-coated with surface proteins or agonistic antibodies are good options. These approaches reduce a 3D complex structure such a cell-cell IS to only two dimensions (XY), enabling high-resolution imaging techniques such as TIRFM (Huppa and Davis, 2003) and, since stimulation occurs at a homogenous, well-defined Z position, image capture at high spatial resolution becomes feasible. If the imaged cell is flat enough, or the Z dimension-restricted cell setups described above are used, secretory vesicle movement at the XY focus plane is a centripetal convergence toward the cSMAC area and can be conveniently imaged and analyzed (Fooksman et al., 2010; Sinha et al., 2016). Using this technique for some structures contained and reorganizing within the IS (i.e., F-actin), the images obtained by using anti-TCR-coated coverslips and lipid bilayers and TIRFM and TIRFM-SIM combination probably exhibit, by far, the highest definition and spatial resolution obtained to date (Murugesan et al., 2016; Sinha et al., 2016). The opportunity to change the composition of the lipid bilayer or the stimulatory antibodies by loading antigens, accessory molecules, changing lipids, etc., allows for reconstitution approaches, increasing the flexibility of this strategy (Huppa and Davis, 2003), although centrosomal area F-actin imaging in living cells will require to develop alternative strategies such as LLSM combined with super-resolution imaging techniques (i.e., STED, 3D-SIM), harboring higher temporal resolution and full competence in the Z dimension.

Apart of the described role of F-actin regions and SMACs in vesicle secretion obtained by high-resolution microscopy, emerging evidences obtained thanks to high-resolution live imaging microscopy support that F-actin-driven and maintained structures such as T cell microvilli, acting as finger-like membrane protrusions or invadosome-like protrusions, may participate in sensing pMHC on APCs, acting as bona fide “synaptosomes” (Sage et al., 2012; Kim et al., 2018; Kim and Jun, 2019), or acting as interfacial protrusions at the IS contact area to facilitate lytic granule secretion and CTL activity (Tamzalit et al., 2019). The formation, maintenance and activity of the later protrusions, as SMACs architecture and functions, both depend on WASP and ARP2/3 activity. The fact that some of the IS actin networks consist of dense actin foci related to protrusive structures rich in F-actin, called invadosome-like protrusions (ILPs) (Hammer et al., 2018; Blumenthal and Burkhardt, 2020), supports that these protrusions should also be analyzed in the IS and actin cytoskeleton studies. Moreover, T lymphocyte microvilli should also be considered not only as structures involved in surveying antigen on APCs or target cells, but also in signaling to APCs or target cells. Thus, these structures are related with relevant immune regulation mechanisms previously discovered (Kim and Jun, 2019). More research involving state-of-the-art microscopy techniques is necessary to understand the mechanisms controlling their generation and function.

## Concluding Remarks

Cells precisely control the formation and the dynamics of both tubulin and actin cytoskeleton networks to coordinate important processes, including motility, cell division, endocytosis and polarized secretion. In addition, cells coordinate the formation of distinct F-actin networks from a general cytosolic pool of actin monomers (Suarez and Kovar, 2016). The available literature concerning the centrosomal subcellular localization and actin cytoskeleton dynamics described here and elsewhere (Dogterom and Koenderink, 2019) demonstrate the existence of a relevant connection between tubulin and actin cytoskeletons and centrosome/MVB polarized traffic and function. Although these links between F-actin and microtubule dynamics are intriguing, very little is known about their molecular bases and functional relevance (Le Floc'h and Huse, 2015). In addition, recent evidences demonstrate that the different F-actin networks appear to be co-ordinately regulated and interconnected. Close coordination between centrosome and centrosomal area F-actin with synaptic F-actin could facilitate the efficient organization of synaptic responses in space and time (Tamzalit et al., 2020). In addition, inhibitor studies indicate that the four discrete cortical actin networks more recently described at the IS (Section Signals Regulating Cortical Actin Reorganization In The Immune Synapse) largely function independently of one another, although there is some coordinate control due to competition for free actin monomer (Hammer et al., 2018). How these distinct cortical and non-cortical networks are regulated, how they interact with the TCR signaling network, as well as their interconnections constitute an intriguing and challenging issue to be addressed in the future. The application of super-resolution microscopy in this context will enable, together with conventional biochemistry techniques, to tackle some of these issues through directly analyzing the interactions between the cytoskeletons and other cell proteins at immune synapses.

## Author Contributions

VC and MI: conceived the manuscript, writing of the manuscript, approved its final content, conceptualization, and writing—review and editing. MI: writing original draft preparation. Both authors contributed to the article and approved the submitted version.

## Conflict of Interest

The authors declare that the research was conducted in the absence of any commercial or financial relationships that could be construed as a potential conflict of interest.

## Abbreviations

3D-SIM: 3D structured illumination microscopy
ADAP: adhesion and degranulation-adaptor protein
APC: antigen-presenting cell
BCR: B-cell receptor for antigen
^C^: center of mass
cSMAC: central supramolecular activation cluster
CTL: cytotoxic T lymphocytes
DAG: diacylglycerol
DGKα: diacylglycerol kinase α
DGKζ: diacylglycerol kinase ζ
Dia1: Diaphanous-1
dSMAC: distal supramolecular activation cluster
F-actin: filamentous actin
FMNL1: formin-like 1
ILPs
invadosome-like protrusions
IS: immune synapse
ITAM: immunoreceptor tyrosine-based motifs
LAT: linker activation of T cells
LFA-1: lymphocyte function-associated antigen 1
LLSM: lattice light sheet microscopy
MHC: major histocompatibility complex
MVB: multivesicular bodies
MTOC: microtubule-organizing center
NK: natural killer
NPFs: actin nucleation promoting factors
PCM: pericentriolar material
PKC: protein kinase C
PKCθ: protein kinase C θ isoform
PKD: protein kinase D
PLC: phospholipase C
PKCδ: protein kinase C δ isoform
pSMAC: peripheral supramolecular activation cluster
ROI: region of interest
SEE: staphylococcal enterotoxin E
SIM: structured illumination microscopy
SL: secretory lysosomes
SLP76: SH2 domain-containing leukocyte protein of 76 kDa
SMAC: supramolecular activation cluster
STED: stimulated emission depletion
TCR: T-cell receptor for antigen
Th: T-helper
TIRFM: total internal reflection fluorescence microscopy
WASH
Wiskott-Aldrich syndrome protein and SCAR homolog
WASp: Wiskott-Aldrich syndrome protein
ZAP70: Syk-kinase zeta chain-associated protein of 70 kDa.
